# Use of Intrathecal Fluorescein in Recurrent Meningitis after Cochlear Implantation

**Published:** 2016-05

**Authors:** Swati Tandon, Satinder Singh, Shalabh Sharma, Asish K. Lahiri

**Affiliations:** 1 *Department of Otorhinolaryngology, Sir Ganga Ram Hospital, Delhi, India.*; 2 *Deputy Director, Cochlear Implant Services, Sir Ganga Ram Hospital Delhi, India.*

**Keywords:** Cochlear implant, Inner ear malformations, Intrathecal fluorescin, Recurrent meningitis

## Abstract

**Introduction:**

Congenital anomalies of the cochlea and labyrinth can be associated with meningitis and varying degrees of hearing loss or deafness. Despite antibiotics, meningitis remains a life threatening complication.

**Case Report:**

We report a case of recurrent meningitis following episodes of otitis media in a cochlear implantee child with bilateral vestibulocochlear malformation, due to fistula in the stapes footplate. Intrathecal fluorescin was used to identify the leak site.

**Conclusion:**

Recurrent meningitis can indicate for possible immunological or anatomical abnormalities as well for chronic parameningeal infections. Intraoperative use of intrathecal fluorescin is an ideal investigative tool to demonstrate cerebrospinal fluid (CSF) leak site in patients in whom other investigations fail to do so.

## Introduction

Recurrent meningitis is described as two or more episodes of meningitis due to different bacteria or, second or further episodes due to the same organism after a period of full recovery from the previous episode (1).

Recurrent meningitis, particularly in children, should alert the physician to the possibility of fistulous communication between the pneumatic cavities of the head and subarachnoidspace. 

With the increasing interest in cochlear implantation, more children with congenital cochlear malformations are being considered for implantation with encouraging results (2). If an implanted child with an inner ear abnormality develops meningitis, one must look for an associated inner ear abnormality as the cause for meningitis. In our case report, recurrent meningitis in a cochlear implantee child with bilateral congenital inner ear abnormality is discussed.

## Case Report

**Fig 1:**
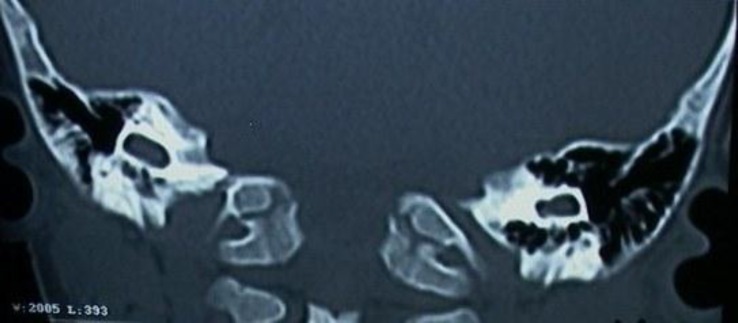
HRCT temporal bone showing an irregular single cavity on the lateral aspect of internal auditory canal bilaterally. No cochlear turns could be identified

A one-year-old male child presented to the clinic after his parents observed that he did not respond to sound. The child had been delivered by caesarian due to complications such as the cord being around the neck and foetal bradycardia. He cried immediately after birth. There was no history of jaundice or meningitis in the postnatal period. The child had mildly delayed physical developmental milestones. Immunisation status was age appropriate.Audiological testing revealed bilateral absent responses on Otoacoustic Emissions (OAE) and Brainstem Evoked Response Audiometry (BERA). Auditory Steady State Response (ASSR) revealed bilateral profound hearing loss. A High Resolution Computed Tomographic (HRCT) scan of the temporal bone revealed an irregular single cavity on the medial aspect of the internal auditory canal bilaterally. No cochlear turns could be identified. Modiolus was absent (Fig.1). 

**Fig2:**
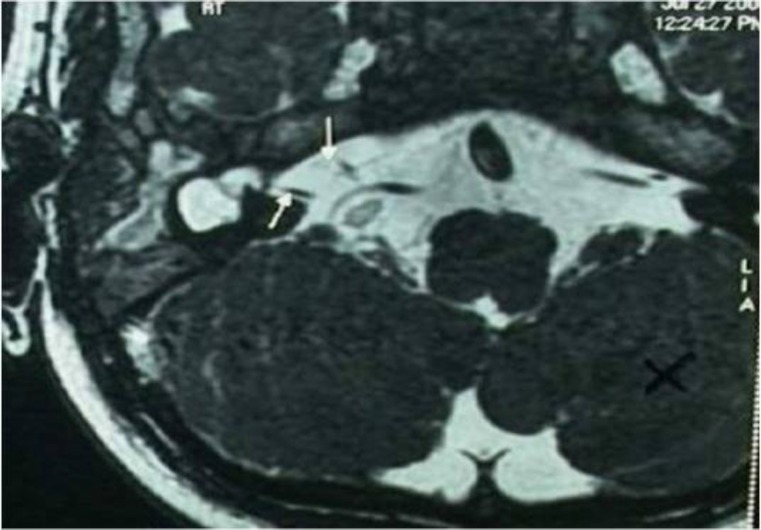
MRI brain showing an absent vestibulo-cochlear nerve complex on the left side and partial visualization on the right side

There was a hypoplastic superior semicircular canal bilaterally. The cavity on the right side appeared larger than the left. Magnetic resonance imaging (MRI) of the brain performed with Constructive Interference in Steady State (CISS) 3D study for evaluation of inner ear structures, revealed an absent vestibulo-cochlear nerve complex on the left side and partial visualization on the right side (Fig.2).

Diagnosis for a common cavity was made. His parents were counseled regarding the possibility of poor auditory response after implantation. The child was vaccinated with pneumococcal, meningococcal, and Hemophilus influenza B vaccine. He was implanted on the right side in September 2006 using the standard postaural incision with posterior tympanotomy approach. Intraoperatively, stapes was found to be hypoplastic, and round window could not be identified. 

Nucleus freedom implant CI24RE (ST) with straight electrode array was inserted through a slit like opening made in the prominence of the common cavity, which was seen in the region of the Lateral semicircular canal (LSCC). CSF leak occurred through the opening in the common cavity. Ten stiffening rings remained outside the opening. Intraoperatively, X ray revealed a satisfactory placement of the active electrodes in contact with the outer wall of the common cavity. Post operatively, his recovery was uneventful. Four months later (Jan 2007), the child presented to his paediatrician with fever and vomiting after an attack of upper respiratory tract infection. A diagnosis for meningitits was confirmed after CSF analysis. Streptococcus nonhemolyticus was isolated on CSF culture. As per the antibiotic sensitivity report, the child was administered intravenous linezolid for 28 days and recovered completely. Four months later, in May 2007, the child once again developed symptoms of meningitis and streptococcus pneumoniae, which was isolated on CSF culture. This episode of meningitits was also preceded by an upper respiratory tract infection. The child received intravenous ceftriaxone for 21 days. After recovery, CT Cisternography of the temporal bone did not reveal any demonstrable leak or communication. In August 2007, the child underwent surgical exploration under general anesthesia to look for a fistulous communication. No site of leakage could be identified. Fibrin glue with soft areolar tissue was applied around the electrode array at the cochleostomy site, upon assumption that this was to be the site. Following this procedure, the child was symptom free for 8 months. However, in April 2008, he developed symptoms for meningitis, preceded by an upper respiratory tract infection. CSF analysis revealed raised protein and reduced sugar with a total leucocyte count of 490/ cmm with 78% polymorphs. The child received intravenous ceftriaxone and vancomycin for 7 days. He recovered well and then presented to us for examination.

On otoscopic examination, a clear fluid level was seen behind the intact tympanic membrane on theleft side with his head upright, which disappeared when the child lied in the supine position. Impedance audiometry revealed type B curve bilaterally. CT Cisternography did not reveal any CSF leak. The implanted ear was re-explored under general anesthesia. Intrathecally, 5% sodium fluorescin dye diluted with CSF was injected and after the child spent an hour in the tredelenburg position with increasing end expiratory pressure, a definite leak was seen in the stapes footplate region between the crura suggesting dehiscence in the footplate.

It is important to note that the footplate was not visible because of the obliquely placed and hypoplastic stapes and overhanging facial canal. Attempt was made to plug the leak in the footplate by soft tissue but the space between the crura was too small. The incus was then removed, stapedius tendon cut, and stapes dislocated and removed. Microscopic examination revealed a circular opening in the footplate. Oval window was packed with soft tissue, gelfoam, and fibrin glue. Five years later, the child is doing well and using his implant.

## Discussion

Development of the inner ear begins early during embryogenesis and by the end of the eighth week, the membranous labyrinth assumes its characteristic convoluted shape. Most inner ear malformations arise when the formation of the membranous labyrinth is interrupted during the first trimester of pregnancy. This interruption may be either a result of inborn genetic error or a consequence of a teratogenic exposure.

In 1791, Carlos Mondini described a membranous and bony dysplasia of the inner ear in a congenitally deaf child (3). Inner ear malformations can be divided into following subgroups: cochlear, vestibular, semicircular canal, internal auditory canal, vestibular, and cochlear aqueduct abnormalities. Cochlear malformations are classified as a common cavity deformity, Michel deformity, cochlear aplasia, cochlear hypoplasia and incomplete partition deformities (4). From the inner ear, CSF can leak through a defect in the otic capsule in the middle ear. The most common site of leakage is the footplate. In literature, defects have been described involving the semicircular canals, vestibule, endolymphatic duct and sac, ossicular chain, facial canal, round window, oval window, and internal auditory canal(5). Various radiological investigations have been used for preoperative localization of CSF leaks such as CT Cisternography, MRcisternography, and high resolution CT scan of the temporal bone.

For many years, CT cisternography has been considered to be the gold standard for detecting CSF leaks. Its detection rate ranges between 40 and 92%; however, the leak must be active (6). The risk of radiation exposure is the major disadvantage of this procedure, especially in children. MR Cisternography typically involves heavily T2-weighted fast spin echo sequences with fat suppression and subtraction of the adjacent background tissue signal to enhance conspicuity of the fistulous tract, or CSF column. It is a noninvasive and non-ionizing technique that can localize the actual site of a fistulous tract. In our patient, MR was not preferred due to the implant in situ.

Intrathecal fluorescin has been used to demonstrate the site of perilymph fistulas in patients with multiple episodes of meningitis. In our patient, preoperative CT cisternography was not able to demonstrate the site of the leak but intraoperative intrathecal fluorescin helped us identify this site. Headache, paresthesis, seizures and vomiting are its side effects but none of them are permanent. In an Italian multicentre studyon intrathecal fluorescin (7), they concluded that side effects of intrathecal fluorescin occur due to error in dosage rather than fluorescin itself. They recommended that the dose of fluorescein should be equal to, or lower than, 1 ml in a 5% mixture (a total of 50 mg of sodic fluorescein). Severe complications like epileptic crises, **grand mal** epilepsy, opisthotonos, peripheral nerve palsy are always related to the direct irritant action of fluorescein by way of chemical meningeal trauma due to overdose of the stain. In a study by LueAJ (8), intrathecal fluorescin was used to identify a CSF leak in a patient with recurrent meningitis caused by mondini dysplasia. In another study by Woolley et al (9), fluorescin dye was used to demonstrate a CSF leak in a patient with a post cochlear implantation who presented with meningitis. In another study (10), intrathecal fluorescin was used to differentiate between CSF otorrhea and rhinorrhea in a patient with recurrent meningitis. Thus, intraoperative use of intrathecal fluorescin is an ideal investigative tool to demonstrate CSF leak site in patients in which other investigations fail to do so.

Our recommendation is that while using intrathecal fluorescin intraoperatively, it is important to perform certain provocative measures to render subtle perilymph flows more visible. These include using the tredelenburg position, raising end expiratory pressure, and waiting for an adequate period of time, which may extend up to an hour or longer, to allow time for the dye to leak from the defect. If the site of the leak cannot be visualized intraoperatively through the posterior tympanotomy, then an anterior tympanotomy should be performed for better visualization of oval and round window areas.

Surgical treatment require repair of fistula between the subarachnoid space and middle ear. Obliteration of the vestibule using the multilayer technique including pieces of muscle, fascia, fibrin glue, and reinforced by a pedicled temporalis muscle graft has been recommended (11). Obliteration of the middle ear and blind sac closure of the external auditory canal has also been recommended. 

Craig et al reported that surgical repair of a fistula in the stapes footplate with fat and soft tissue in a patient with mondini dysplasia was not successful in the first stage (12). They concluded that bony closure of the fistula is essential to prevent failure. In our patient, the oval window opening was plugged using soft tissue and fibrin glue without any episode of meningitis since the last 5 years, therefore, we recommend that correct identification of site of leakage and meticulous repair is essential to reduce failure rates. Our experience in many cases of CSF rhinorrhea repair also suggests that bony closure is not essential for a successful repair.

For those candidates with an inner ear malformation without a CSF leak or meningitis, who are undergoing cochlear implant surgery, we suggest that potential sites of CSF leakage be closed by soft tissue packing. Careful examination of both the oval and round windows at the time of implantation is essential to look for any fistulous opening or CSF leakage. The presence of deformed stapes should also be noted. In the absence of any obvious leak, oval and round window may be sealed as a preventive measure.

Meningitis following cochlear implantation is a difficult problem. Therefore, evaluation and intervention must be performed meticulously. The implanted ear is the entry site for meningitis in most of the cases reviewed in literature. However, the non implanted ear must also be evaluated carefully in a patient with bilateral inner ear malformation. Melek Kezban Gurbuz et al^11^ have reported a case of recurrent meningitis in a 8-year-old cochlear implantee due to a fistula in the stapes footplate in the non implanted ear.

Follow-up, intense counselling with the parents who are taught to recognize the early signs of middle ear infection and meningitis, avoidance of head trauma, and vaccination is recommended. Surgical management of patients with CSF leak requires definitive identification of the anatomic source. In our case, the first surgical procedure was exploratory as CSF leakage was not demonstrated with radiological testing. Accurate localization preoperatively enhances the chance of successful identification of the site of CSF fistula, thereby minimizing the chance of negative or repeated explorations. CT cisternography has been commonly used for preoperative localization of the site of leakage, but in our case it was not successful.

## Conclusion

Therefore, intraoperative use of intrathecal flourescin should be considered as an important diagnostic tool in these cases. Measures should be taken to seal potential sites of CSF leaks at the time of implantation in patients undergoing cochlear implant surgery with inner ear malformations to prevent future meningitis. Intraoperative use of intrathecal fluorescin when CT or MR cisternography fails to identify the site of leakage with appropriate measures for identification and repair of the fistula site is recommended.
